# The prevalence and outcomes of depression in older HIV-positive adults in Northern Tanzania: a longitudinal study

**DOI:** 10.1007/s13365-023-01140-4

**Published:** 2023-05-25

**Authors:** Damneek Dua, Oliver Stubbs, Sarah Urasa, Jane Rogathe, Ashanti Duijinmaijer, William Howlett, Marieke Dekker, Aloyce Kisoli, Elizabeta B. Mukaetova-Ladinska, William K. Gray, Thomas Lewis, Richard W. Walker, Catherine L. Dotchin, Bingileki Lwezuala, Philip C. Makupa, Stella Maria Paddick

**Affiliations:** 1 Newcastle University, Campus for Ageing and Vitality, Westgate Road, NE4 6BE Newcastle upon Tyne, UK; 2 Kilimanjaro Christian Medical University College, Moshi, Kilimanjaro Tanzania; 3 Haydom Hospital Manyara Tanzania, Haydom, Tanzania; 4 Department of Neuroscience, Behaviour and Psychology, University of Leicester, Leicester, UK; 5 Northumbria Healthcare NHS Foundation Trust, North Tyneside General Hospital, North Shields, UK; 6 Cumbria Northumberland Tyne and Wear NHS Foundation Trust, Newcastle upon Tyne, UK; 7 Mawenzi Regional Referral Hospital, Moshi, Kilimanjaro Tanzania

**Keywords:** HIV-associated neurocognitive disorders, Sub-Saharan Africa, Depression, Tanzania, Older adults

## Abstract

Studies of depression and its outcomes in older people living with HIV (PLWH) are currently lacking in sub-Saharan Africa. This study aims to investigate the prevalence of psychiatric disorders in PLWH aged ≥ 50 years in Tanzania focussing on prevalence and 2-year outcomes of depression. PLWH aged ≥ 50 were systematically recruited from an outpatient clinic and assessed using the Mini-International Neuropsychiatric Interview (MINI). Neurological and functional impairment was assessed at year 2 follow-up. At baseline, 253 PLWH were recruited (72.3% female, median age 57, 95.5% on cART). DSM-IV depression was highly prevalent (20.9%), whereas other DSM-IV psychiatric disorders were uncommon. At follow-up (*n* = 162), incident cases of DSM-IV depression decreased from14.2 to 11.1% (χ^2^: 2.48, *p* = 0.29); this decline was not significant. Baseline depression was associated with increased functional and neurological impairment. At follow-up, depression was associated with negative life events (*p* = 0.001), neurological impairment (*p* < 0.001), and increased functional impairment (*p* = 0.018), but not with HIV and sociodemographic factors. In this setting, depression appears highly prevalent and associated with poorer neurological and functional outcomes and negative life events. Depression may be a future intervention target.

**Supplementary Information:**

The online version contains supplementary material available at 10.1007/s13365-023-01140-4.

## Introduction

There are over 38 million people living with HIV (PLWH) worldwide (UNAIDS 2019). New infections total 1.7 million annually, two thirds of which occur in sub-Saharan Africa (SSA) (UNAIDS 2019). Rapidly expanding access to combined antiretroviral therapy (cART) has markedly increased life expectancy of PLWH. In Africa, the population aged ≥ 50 is expected to triple by 2030 from 74.4 to 235.1million (UNAIDS 2014). SSA currently accounts for 60% of all PLWH over the age of 50 (UNAIDS 2014). This newly emergent ageing population brings new challenges in the form of chronic HIV-associated comorbidities.

Mental disorders (primarily depression) are predicted to become the leading cause of disability worldwide by 2030, with over 322 million people currently affected (Bing et al. 2001; Kang et al. 2015). Prevalence data for depression in SSA are currently limited. Based on limited available evidence, an estimated 4% of the adult community population of the WHO Africa region meet criteria for depression, with substantially higher rates in small hospital-based studies (Gbadamosi et al. 2022). Globally, the rates of depression in PLWH appear to be 2–4 times higher than the general population (Bernard et al. 2017) with an estimated pooled prevalence of 9–32% reported in PLWH in SSA. This is important, because depression is well-recognised to negatively impact HIV disease outcomes (in SSA and elsewhere) resulting in treatment failure, higher HIV viral load, and increased mortality.

The pathophysiology of depression in HIV is not well understood. Current hypothesised aetiologies include biological and psychosocial pathways (Nanni et al. 2015). The biological pathway attributes depression to persistent viral presence in the central nervous system (CNS), thus acting as a reservoir (Nanni et al. 2015). This prolongs immunological activation and releases toxic viral proteins and inflammatory cytokines which results in depression as part of a spectrum of neurological impairment due to the resultant neuronal damage (Nanni et al. 2015). This links with the increasingly recognised ‘inflammatory’ hypothesis of depression aetiology in conditions other than HIV, as well as in HIV infection (Mudra Rakshasa-Loots et al. 2022; Osimo et al. 2019; Bell et al. 2017). The psychosocial pathway attributes depression to the psychological burden of living with a chronic, disabling, and stigmatised disease (Nanni et al. 2015), which is particularly pertinent in HIV. Therefore, both biological and psychosocial stressors are likely to be highly relevant in the aetiology of depression in PLWH. Depression and HIV may also have a bidirectional relationship in that depression appears to be both a risk factor for and consequence of HIV infection (Goin et al. 2020). An additional challenge is that depression may be associated with individual cART medications (e.g. efavirenz) and with common comorbidities (particularly tuberculosis (TB)) (Munoz-Moreno et al. 2009; Sweetland et al. 2017).

Despite the newly ageing HIV population on cART, few studies have investigated depression in older PLWH. These studies, appraised in the discussion, mostly use cross-sectional data and longitudinal data are lacking (Rabkin et al. 1997; Johnson et al. 1999; Olley et al. 2006; Rodkjaer et al. 2011; Orlando et al. 2002) (see supplementary Table 4). In addition, existing SSA longitudinal studies of depression in HIV focus almost exclusively on younger populations and/or specific higher risk groups, such as intravenous drug users, marginalised groups, and untreated populations(Antelman et al. 2007; Marwick and Kaaya 2010). Current data, therefore, lack applicability to the cART-treated ageing population rapidly increasing in SSA.

Older PLWH may be at specific increased risk of neurological impairment and disability. For example, HIV-associated neurocognitive disorders (HAND) affect up to 50% of older PLWH worldwide (Heaton et al. 2010). Since depression is a well-recognised risk for and consequence of neurodegenerative dementias (Cipriani et al. 2015), data on neurocognitive outcome of depression in older PLWH is needed and currently lacking.

In summary, depression and HIV are predicted to become the first and second leading causes of disability worldwide by 2030 resulting in huge potential individual and societal consequences for those affected (Bing et al. 2001; World Health Organisation 2020). There is a critical gap in current knowledge regarding depression in older PLWH, potentially modifiable aetiological factors and outcomes. Without assessment of the longer-term effect of depression, progress in low-resource settings may not be prioritised due to lack of evidence to inform policy makers.

We therefore aimed to:

1. Estimate prevalence and risk factors of depression, in the context of other psychiatric disorders, in older PLWH in Tanzania.
2. Estimate longitudinal prevalence of depression and its neurological and functional outcomes in PLWH aged ≥ 50 years receiving standard HIV clinic follow-up.
3. Explore the relationship of depression to the biological and psychological hypothesised aetiological pathways.

## Methods

### Setting

The study took place at Mawenzi Regional Referral Hospital (MRRH) HIV Care and Treatment Centre (CTC) in the Kilimanjaro region of Northern Tanzania. This clinic is a government-run, free-of-charge service. The estimated national prevalence of HIV infection in Tanzania is 4.6% with 71% of those aware of their HIV status receiving treatment (UNAIDS 2018). At baseline (2016), 820 PLWH aged ≥ 50 years were registered with the MRRH clinic. MRRH has a psychiatric clinic, though a trained psychiatrist (responsible for the entire Kilimanjaro region) was recruited only in 2018. Currently, Tanzania has only 0.52 qualified mental health workers per 100,000 population (World Health Organisation 2017).

### Ethics

Ethical approval was granted by the Tanzanian National Institute of Medical Research and Kilimanjaro Christian Medical College Research Ethics Committee (No. 896). Verbal and written information was given to each participant before informed consent was obtained. If participants lacked capacity to consent, assent was sought from a close relative. Consent was rechecked at follow-up. The study protocol included locally agreed referral pathways for individuals diagnosed with significant psychiatric disorders.

### Recruitment, sampling, and background data

The primary purpose of the baseline study was identification and screening of HIV-associated neurocognitive disorders (HAND) by the American Academy of Neurology (AAN) criteria (Antinori et al. 2007). A detailed description of recruitment and baseline assessment has previously been published (Kellett-Wright et al. 2021). In brief, a systematic sample (every third eligible individual) was recruited from March to May 2016. Inclusion criteria were individuals aged ≥ 50 years attending routine follow-up. Those attending emergency appointments too physically unwell to comfortably participate or newly diagnosed were excluded.

Individuals included at baseline were offered annual follow-up to coincide with routine clinic appointments during the annual study follow-up (resource-limited to March to May annually). Longitudinal analysis was limited to individuals completing follow-up evaluation between March and May 2018. Detailed HIV disease severity, e.g. CD4 count or viral load, and comorbidity data were obtained from standardised clinic records as previously reported (Kellett-Wright et al. 2021). Other demographic data were self-reported and corroborated by informants where necessary.

### Assessment of psychiatric disorders

Baseline screening and diagnosis of depression and other psychiatric disorders were completed by a doctorate-level specialist nurse and specialist nurse with experience of older person’s mental health research (JR, AK). A local translation of the 15-item Geriatric Depression Scale (GDS) was used to screen for depression. The GDS-15 was selected despite cultural limitations as it is widely used in SSA, including in large epidemiological studies (Sarfo et al. 2017; Sokoya and Baiyewu 2003), and no robustly validated alternative currently exists. The GDS translation used had undergone forward and backward translation by local clinicians experienced in mental health care. A cut-off of 5/15 (previously used for ICD-10/DSM-IV depression in SSA) was applied. The GDS was interviewer-administered, an accepted method in low-literacy settings(Almeida and Almeida 1999). Psychiatric disorders were identified using a local translation of the Mini-International Neuropsychiatric Interview (MINI), a widely used structured ‘stem and leaf’ tool which utilises the DSM-IV criteria and is validated for depression screening in PLWH in SSA (Tsai 2014).

### Neurological impairment and HIV-associated neurocognitive disorder

Neurological symptoms were self-reported by structured questionnaire (supplementary Table 1) and included memory, thinking, balance impairment, subjective slowness, and loss of feeling in the hands/feet.

HIV-associated neurocognitive disorders (HAND) by AAN (2007) criteria were classified by consensus panel (SMP, EML, RA, TL). Full HAND assessment (details previously published (Eaton et al. 2020)) included locally normed low-literacy neuropsychological test battery, structured mental state examination, abbreviated neurological examination, and creation of summary case notes for consensus panel review. As per AAN criteria, HAND were not diagnosed if another more likely neurological or psychiatric cause (including severe depression) was identified.

### Functional impairment

Subjective functional impairments (employment/home responsibilities) were self-reported (supplementary Table 1**)**. Informant collateral history to confirm/refute cognitive or functional impairment was obtained, usually from a close relative (by telephone where necessary), with participants’ consent. Informants were told this was a general ageing study and HIV was not mentioned to protect patients’ confidentiality. Overall clinician rating of function was recorded using the Karnofsky performance scale (0–100%) (Peus et al. 2013) widely used in HIV settings. This rating considered self-reported impairment, clinical assessment, and collateral history.

### Follow-up assessment

Follow-up assessment (2018) was similar to baseline, with the following changes. (1) All individuals were screened with the GDS-15 and, only if screen positive (≥ 5/15), the DSM-IV depression element of the MINI completed (AK). Other DSM-IV psychiatric disorders were not evaluated due to very low prevalence of disorders other than depression at baseline and to avoid participant fatigue. (2) At follow-up, participants were asked if they had experienced any significant negative life event in the previous 2 years (e.g. bereavement, significant loss, or other negative life event) to ensure these issues were not missed. (3) HIV viral load measurement became locally available in 2017 (following a change in national guidelines) and was included as a HIV disease severity outcome at follow-up. Viral suppression was defined as ≤ 20 copies/ml.

### Statistical analysis

Data analysis was supported by IBM SPSS (version 26; IBM, Armonk, NY, USA). Standard descriptive statistics (e.g. mean, median, standard deviation (SD), interquartile range (IQR), and frequency) and inferential tests (e.g. chi-squared, Mann–Whitney *U*, and *t* test) were used for group comparisons depending on the level and distribution of the data. All data analyses were two-tailed at a 5% significance level. Only those with full data at both time points were included in longitudinal analysis (253 and 162 participants in 2016 and 2018, respectively).

## Results

Of 253 individuals with complete data at baseline 2016, 162 (64.0%) were followed up in 2018 (Table 1). Demographic, HIV disease, and comorbidity characteristics at baseline are summarised in supplementary Table 2 and are also previously published (Kellett-Wright et al. 2021; Eaton et al. 2020). The majority were female (72.3%), educational level was low (64% completed primary education), and current HIV disease control was good (95.5% cART-treated, mean CD4 526.5 mm/l), though most (60%) had advanced disease (WHO stage 3 or 4).

**Table 1:** Prevalence of DSM-IV psychiatric disorders and self-reported neurological impairment in the baseline cohort (*n* = 253)

	**Prevalence (*n*, %)**	**95% confidence interval**
**Psychiatric disorder**		
** Major depressive disorder**	24 (9.5%)	5.9–13.1
** Dysthymia/depression** (missing = 3)	42 (16.6%)	12.4–21.6
** Mania**	3 (1.2%)	−0.1–2.5
** Hypomania** (missing = 2)	0	0
** Panic disorder** (missing = 30)	2 (0.8%)	−0.3–1.9
** Agoraphobia + panic** (missing = 1)	0	0
** Agoraphobia + no panic** (missing = 29)	1 (0.4%)	−0.4–1.2
** Social phobia** (missing = 1)	6 (2.4%)	0.5–4.3
** Obsessive compulsive disorder**	1 (0.4%)	−0.4–1.2
** Post-traumatic stress disorder**	2 (0.8%)	−0.3–1.9
** Alcohol dependence**	1 (0.4%)	−0.4–1.2
** Substance abuse**	0	0
** Mood disorder/psychotic features** (missing = 1)	4 (1.6%)	0.0–3.1
** Any psychotic illness** (missing = 1)	1 (0.4%)	−0.4–1.2
** Generalised anxiety disorder** (missing = 1)	3 (1.2%)	−0.1–2.5
** Antisocial personality disorder** (missing = 1)	0	0
** Summary of psychiatric disorders**		
** Any mood disorder**	46 (18.2%)	13.4–22.9
** Any anxiety disorder**	10 (4.0%)	1.6–6.4
** Any psychotic disorder**	7 (2.8%)	0.7–4.8
** Any alcohol disorder**	0	0
** Psychiatric medications** (amitriptyline)	7 (2.8%)	0.75–4.79
** Self-reported neurological/functional impairment**		
** Balance issues** (missing = 7)	30 (11.9%)	7.9–15.8
** Slow hand movements** (missing = 8)	27 (10.7%)	6.9–14.5
** Neuropathy** (missing = 8)	90 (35.6%)	29.7–41.5
** Headaches** (missing = 7)	90 (35.6%)	29.7–41.5
** Normal task difficulties** (missing = 10)	66 (26.1%)	20.7–31.5
** Home responsibilities difficulties** (missing = 10)	17 (6.7%)	3.6–9.8
** Summary of self-reported neurological/functional impairment**		
** Functional impairment** (missing = 5)	69 (27.3%)	21.8–32.8
** Neurological symptoms** (missing = 5)	112 (44.3%)	38.1–50.4
** Clinician reported variables**		
** HAND diagnosis**	119 (47.0%)	40.9–53.2

For demographic and HIV disease-specific data, see Supplementary online data (16, 21)

### Depression and other psychiatric disorders at baseline (2016)

Affective and non-affective psychiatric disorders by DSM-IV MINI criteria are listed in Table 1. Depression was highly prevalent by both GDS-15 (53, 20.9%) (Table 4) and MINI DSM-IV criteria (42, 16.6%) (Table 1). Few were treated with psychiatric medication (DSM-IV *n* = 2 (3.8%), GDS-15 *n* = 3 (7.1%) prescribed low-dose amitriptyline). Other psychiatric disorders (anxiety disorders, psychotic disorders) were uncommon. Alcohol dependence was reported by one participant, and no participants reported other substance misuse.

### Neurological and functional impairment at baseline (2016)

Neurological and functional impairments were common (Table 1) particularly neuropathy, headaches, and difficulties with home and work tasks. Almost half (47.0%) met AAN HAND criteria.

### Depression at baseline and non-significant outcomes

DSM-IV depression at baseline was not associated with HIV disease severity markers including legacy effect (proxy measure: nadir CD4), current efavirenz prescription or TB treatment, measured sociodemographic factors, or presence of neurocognitive or functional impairments (Table 2).

**Table 2:** Demographic, HIV disease, and neurological and functional impairment factors associated with DSM-IV depression in 2016 baseline cohort (*n* = 253)

**2016 variable**	**DSM-IV depression 2016**	**No DSM-IV depression 2016**	**Test and significance**
**Prevalence**	42 (16.6%)	211 (83.4%)	N/A
**Psychosocial factors**			
** Education** (primary education not completed)	13 (31%)	78 (37.0%)	0.55 (χ^2^) (*p* = 0.46)
** Education** (primary school educated)	29 (69.0%)	133 (63.0%)	
** Lives alone**	9 (21.4%)	34 (16.1%)	1.43 (χ^2^) (*p* = 0.49)
** Unemployed**	4 (9.5%)	25 (11.8%)	0.43 (χ^2^) (*p* = 0.93)
** Mean Karnofsky performance scale** (0–100%)	92.4%	93.9%	933.00 (M-W U) (*p* = 0.77)
**HIV disease factors**			
** Mean CD4**	438.6	530.2	2.00 (*t* test) (*p* = 0.17)
** Mean Nadir CD4**	203.6	196.1	−0.26 (*t* test) (*p* = 0.84)
** Medication adherence** (100% adherence)	23 (54.8%)	152 (72.0%)	5.34 (χ^2^) (*p* = 0.07)
** Efavirenz**	23 (54.8%)	111 (52.6%)	3.07 (χ^2^) (*p* = 0.80)
**Self-reported neurological/functional impairment**			
** Balance problems**	2 (4.8%)	28 (13.3%)	2.48 (χ^2^) (*p* = 0.12)
** Slow hand movements**	3 (7.1%)	24 (22.4%)	0.70(χ^2^) (*p* = 0.40)
** Neuropathy**	11 (26.2%)	79 (37.4%)	2.11 (χ^2^) (*p* = 0.15)
** Headaches**	25 (59.5%)	136 (64.5%)	0.47 (χ^2^) (*p* = 0.49)
** Normal task difficulties**	10 (23.8%)	56 (26.5%)	0.12 (χ^2^) (*p* = 0.72)
** Home responsibilities difficulties**	2 (4.8%)	15 (7.1%)	0.30 (χ^2^) (*p* = 0.58)
** Summary of self-reported neurological/functional impairment**			
** Functional impairment**	10 (23.8%)	59 (28.0%)	0.31 (χ^2^) (*p* = 0.58)
** Neurological symptoms**	14 (33.3%)	98 (46.4%)	2.44 (χ^2^) (*p* = 0.12)
**Clinician reported variables**			
** HAND diagnosis**	25 (59.5%)	94 (44.5%)	3.15 (χ^2^) (*p* = 0.76)

### Follow-up cohort 2016–2018

A total of 162 individuals were fully assessed at baseline and follow-up. Those not followed up were not necessarily lost to follow-up by the clinic, but the majority (*n* = 71) did not attend during the study follow-up window. Those followed up did not significantly differ in demographic (% female, median age, % primary educated) or HIV disease severity factors (mean CD4, WHO stage 1/2 vs 3/4) from those not followed up (supplementary Table 3). Only a small minority (*n* = 8) had died.

**Fig. 1:**
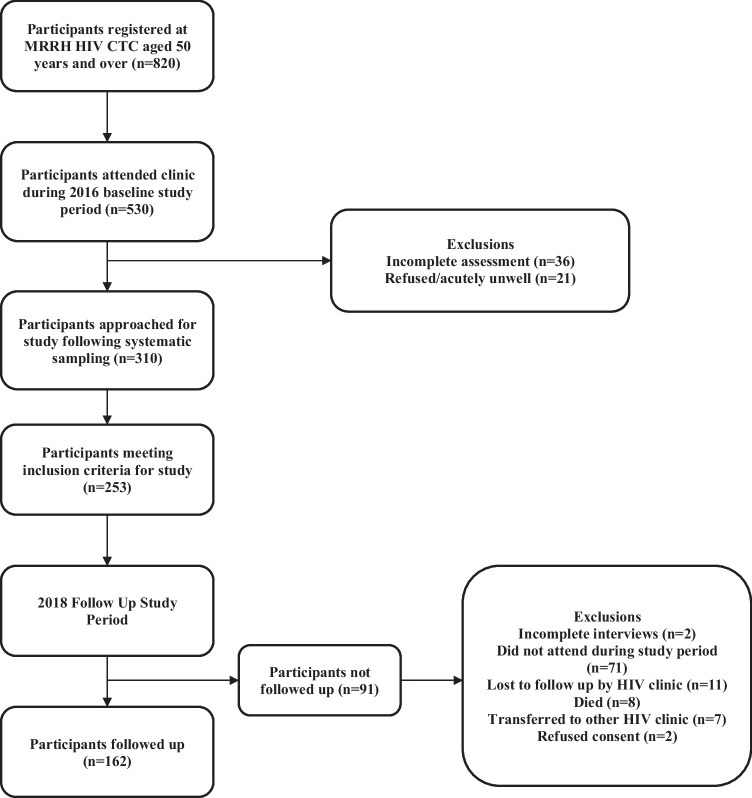
Study flowchart. Those followed up in 2018 did not significantly differ in demographic (%female 70.4 95%CI, χ^2^: 0.87, *p* = 0.35; median age 59 95%CI, *t* test: 0.16, *p* = 0.75; %primary educated 67.9 95%CI, χ^2^: 6.64, *p* = 0.25) or HIV disease severity factors (mean CD4 530.2 95%CI, *t* test: 1.47, *p* = 0.39, WHO stage 1/2 14.8%, WHO stage 3/4 93.2%) from those not followed up

Demographic and HIV disease data at each time point are summarised in supplementary data Table 3. Between baseline and follow-up, GDS-15 depression significantly decreased (20.9% to 13.0% χ^2^: 4.30, *p* = 0.038), but there was a non-significant decrease of depression by DSM-IV criteria (16.6% to 11.1%, χ^2^: 2.41, *p* = 0.12) (supplementary Table 3, Fig. 1).

### Two-year outcome of depression at baseline

Outcomes of those with and without DSM-IV and GDS-15 depression at baseline are outlined in Table 3 and supplementary data Table 4, respectively. Most HIV disease outcomes (CD4, viral suppression, WHO stage, medication adherence) were not significantly different in those with and without depression at baseline. There was a trend of higher prevalence of unsuppressed HIV viral load in individuals with both DSM-IV (43.5% *n* = 10 vs 30.2%, *n* = 43, *p* = 0.45) and GDS-15 depression (41.9%, *n* = 13 vs 29.8%, *n* = 39, *p* = 0.83). The use of efavirenz significantly decreased (supplementary Table 3) (53.0% to 31.5% χ2: 91.30, p<0.001), and those on second line cART treatment increased (Tables 3 and 4).

**Table 3:** Outcomes at follow-up (2018) of DSM-IV depression at baseline (2016)

**2018 variable**	**DSM-IV depression 2016**	**No DSM-IV depression 2016**	**Test and significance**
**Prevalence**	**42 (16.6%)**	**211 (83.4%)**	**N/A**
**Psychosocial outcomes**			
** Life event**	15 (65.2%)	50 (36.0%)	7.65 (χ^2^) (*p* = 0.04)
** Lives alone**	6 (26.1%)	31 (22.3%)	0.40 (χ^2^) (*p* = 0.53)
** Unemployed**	3 (13.0%)	13 (9.4%)	0.28 (χ^2^) (*p* = 0.60)
** Depressed in 2018.** (GDS definition)	7 (30.4%)	14 (10.1%)	7.25 (χ^2^) (*p* = 0.01)
** Depressed in 2018.** (DSM-IV definition)	3 (13.0%)	11 (7.9%)	2.48 (χ^2^) (*p* = 0.29)
** Mean Karnofsky performance status** (0–100%)	88.7%	94.2%	1174.50 (M-W U) (*p* = 0.02)
**HIV disease outcomes**			
** Mean CD4 count** (mm/l)	572.30	522.56	−0.86 (*t* test) (*p* = 0.14)
** Mean Nadir CD4 count** (mm/l)	252.97	190.81	−1.45 (*t* test) (*p* = 0.28)
** Viral load not suppressed**	10 (43.5%)	42 (30.2%)	1.59 (χ^2^) (*p* = 0.45)
** Medication adherence** (100% adherence)	22 (95.7%)	134 (96.4%)	3.39 (χ^2^) (*p* = 0.07)
** WHO stage 1/2**	4 (17.4%)	20 (14.4%)	0.05 (χ^2^) (*p* = 0.83)
** WHO stage 3/4**	19 (82.6%)	108 (77.7%)	
** First-line treatment**	11 (47.8%)	73 (52.5%)	0.22 (χ^2^) (*p* = 0.88)
** Second-line treatment** (missing = 10)	2 (8.7%)	15 (10.8%)	
** Efavirenz** (missing = 54)	7 (50.0%)	44 (46.8%)	1.29 (χ^2^) (*p* = 0.52)
**Self-reported neurological/functional impairment**			
** Memory problems**	10 (43.5%)	41 (29.5%)	2.11 (χ^2^) (*p* = 0.35)
** Concentration problems**	4 (17.4%)	7 (5.0%)	4.76 (χ^2^) (*p* = 0.03)
** Difficulty thinking clearly**	1 (4.3%)	2 (1.4%)	0.92 (χ^2^) (*p* = 0.34)
** Cognitive impairment**	12 (52.2%)	42 (30.2%)	6.18 (χ^2^) (*p* = 0.10)
** Balance problems**	6 (26.1%)	7 (5.0%)	11.85 (χ^2^) (*p* = 0.001)
** Slow hand movements**	7 (30.4%)	8 (5.8%)	14.31 (χ^2^) (*p* < 0.001)
** Neuropathy**	11 (47.8%)	41 (29.5%)	3.04 (χ^2^) (*p* = 0.08)
** Headaches**	8 (34.8%)	32 (23%)	1.47 (χ^2^) (*p* = 0.23)
** Normal task difficulties**	5 (21.7%)	19 (13.7%)	1.018 (χ^2^) (*p* = 0.31)
** Home responsibilities difficulties**	5 (21.7%)	14 (10.1%)	2.60 (χ^2^) (*p* = 0.11)
**Summary of self-reported neurological/functional impairment**			
** Functional impairment**	8 (34.8%)	27 (19.4%)	2.79 (χ^2^) (*p* = 0.25)
** Neurological symptoms**	13 (56.5%)	47 (33.8%)	21.47 (χ^2^) (*p* > 0.001)
**Clinician reported variables**			
** HAND diagnosis**	10 (43.5%)	86 (61.9%)	2.77 (χ^2^) (*p* = 0.10)
** Symptomatic HAND**	3 (13.0%)	29 (20.9%)	0.76 (χ^2^) (*p* = 0.38)

**Table 4:** Outcomes at follow-up (2018) of having depression by GDS criteria at baseline 2016

**2018 data**	**GDS depression at baseline**	**No GDS depression at baseline**	**Test and significance (*p* value)**
**Psychosocial factors**			
** Life event** (missing = 3)	21 (67.7%)	44 (33.6%)	13.03 (χ^2^) (*p* = 0.001)
** Lives alone** (missing = 2)	8 (25.8%)	29 (22.1%)	0.26 (χ^2^) (*p* = 0.61)
** Unemployed**	7 (22.6%)	9 (6.9%)	7.03 (χ^2^) (*p* = 0.03)
** Depressed in 2018** (GDS definition)	14 (45.2%)	7 (5.3%)	35.23 (χ^2^) (*p* < 0.001)
** Depressed in 2018** (MINI definition)	3 (9.7%)	11 (8.4%)	1.73 (χ^2^) (*p* = 0.42)
** Mean Karnofsky performance status** (0–100%)	86.8%	95.0%	1287.50 (M-W U) (*p* < 0.001)
**HIV disease factors**			
** Mean CD4 count** (mm/l)	549.48	525.56	−0.45 (*t* test) (*p* = 0.26)
** Mean Nadir CD4 count** (mm/l)	252.97	190.81	−1.86 (*t* test) (*p* = 0.09)
** Viral load not suppressed**	13 (41.9%)	39 (29.8%)	3.91 (χ^2^) (*p* = 0.14)
** WHO stage 1/2**	5 (16.1%) (missing 2)	19 (14.5%) (missing 9)	0.49 (χ^2^) (*p* = 0.83)
** WHO stage 3/4**	24 (77.4%)	103 (78.7%)	
** Medication adherence** (100% adherence)	25 (80.6%)	112 (85.5%) (missing 3)	0.98 (χ^2^) (*p* = 0.32)
** First-line treatment**	12 (38.7%)	72 (55.0%)	0.13 (χ^2^) (*p* = 0.72)
** Second-line treatment**	3 (9.7%)	14 (10.7%)	
** Efavirenz** (missing = 54)	8 (42.1%)	43 (48.3%)	5.14 (χ^2^) (*p* = 0.08)
**Self-reported neurological impairment**			
** Memory problems**	13 (41.9%)	38 (29.0%)	2.37 (χ^2^) (*p* = 0.31)
** Concentration problems**	4 (12.9%)	7 (5.3%)	2.26 (χ^2^) (*p* = 0.13)
** Difficulty thinking clearly**	1 (3.2%)	2 (1.5%)	0.40 (χ^2^) (*p* = 0.53)
** Cognitive impairment**	15 (48.3%)	39 (29.8%)	5.84 (χ^2^) (*p* = 0.12)
** Balance problems**	7 (22.6%)	6 (4.6%)	11.01 (χ^2^) (*p* = 0.001)
** Slow hand movements**	6 (19.4%)	9 (6.9%)	4.65 (χ^2^) (*p* = 0.03)
** Neuropathy**	14 (45.2%)	38 (29.0%)	3.00 (χ^2^) (*p* = 0.08)
** Headaches**	8 (25.8%)	32 (24.4%)	0.03 (χ^2^) (*p* = 0.87)
** Normal task difficulties**	7 (22.6%)	17 (13.0%)	1.83 (χ^2^) (*p* = 0.18)
** Home responsibilities difficulties**	8 (25.8%)	11 (8.4%)	7.34 (χ^2^) (*p* = 0.01)
**Summary of self-reported neurological impairment**			
** Functional impairment**	12 (38.7%)	23 (17.6%)	6.69 (χ^2^) (*p* = 0.04)
** Neurological symptoms**	16 (51.6%)	44 (33.6%)	11.01 (χ^2^) (*p* = 0.001)
**Clinician reported variables**			
** HAND diagnosis**	17 (54.8%)	79 (60.3%)	0.31 (χ^2^) (*p* = −0.58)
** Symptomatic HAND**	9 (29%)	23 (17.6%)	2.08 (χ^2^) (*p* = 0.23)

### Psychosocial outcomes

Individuals meeting DSM-IV or GDS-15 depression criteria in 2016 were significantly more likely to report negative life events in the previous year at follow-up in 2018 compared to those who did not meet depression criteria at baseline in 2016 (Table 3, supplementary data Table 4). Unemployment at follow-up was associated with baseline depressive symptoms by the GDS-15 (Table 4) but not DSM-IV criteria (Table 3).

### Neurological outcomes

**Fig. 2:**
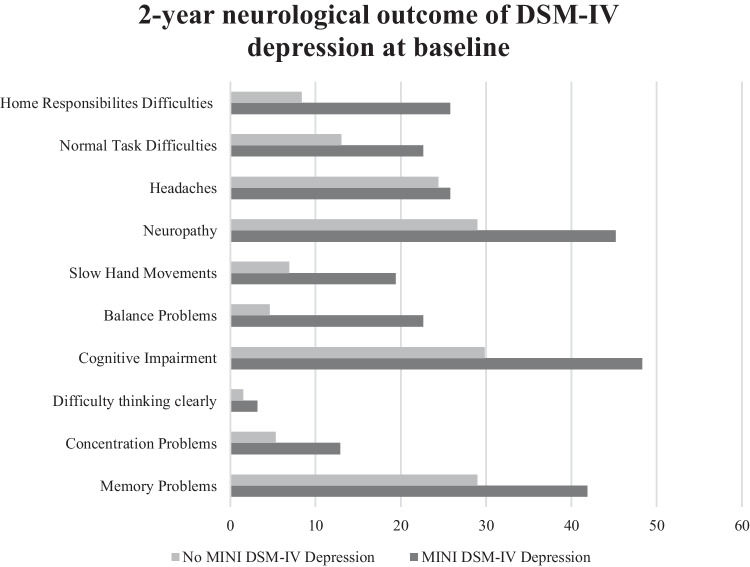
Two-year neurological outcome of MINI DSM-IV depression at baseline (2016)

Self-reported neurological outcomes are illustrated in Fig. 2. Figure 2 shows those depressed at baseline had poorer neurological outcomes at follow-up compared to those not depressed at baseline. Baseline DSM-IV depression was significantly associated with self-reported neurological impairment (impaired concentration, balance, slow hand movements) at follow-up but not a formal HAND diagnosis (Table 3).

### Functional impairment

**Fig. 3:**
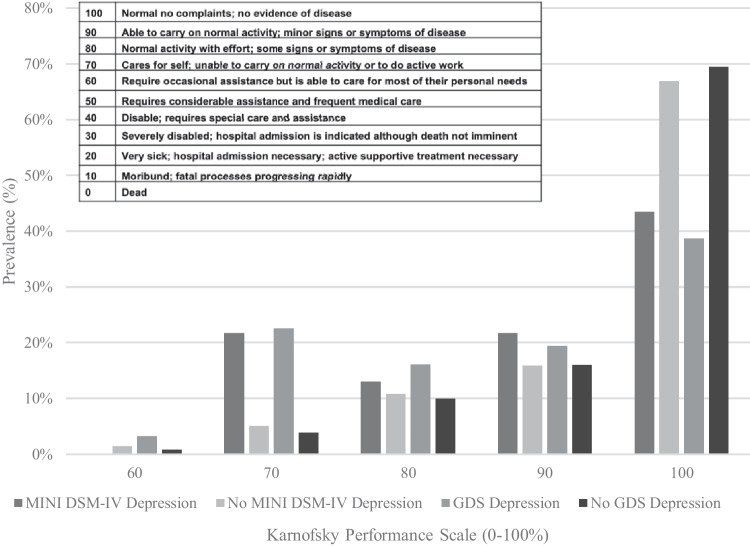
Functional outcome by Karnofsky performance status in those with and without DSM-IV depression and/or depression symptoms GDS ≥ 5/15 at baseline

Figure 3 shows those depressed by both MINI DSM-IV and GDS-15 criteria have poorer functionality by the Karnofsky performance status compared to non-depressed participants. At follow-up, self-reported difficulties with everyday tasks were twice as likely and with home responsibilities three times as likely, in those with depression at baseline (Fig. 2, Table 3). Similarly, over 20% of those with depression at baseline were clinician-rated as substantially functionally impaired (Karnofsky ≤ 70%, indicating need for assistance with daily living activities) compared with ≤ 5% of those without baseline depression (Fig. 3). Prevalence of both HAND and functional impairment increased by 12.3% and 5.1%, respectively, in 2016–2018 though self-reported neurological symptoms decreased by 7.3%.

## Discussion

This study is the first to report longitudinal prevalence of depression in older PLWH in SSA alongside biological, psychosocial, and functional outcomes. We report a high depression prevalence (20.9% DSM-IV, 16.6% GDS-15) but were unable to identify other SSA studies using comparable diagnostic criteria.

One South African study (*n* = 422) reported prevalence of 14.8% by ICD-10 criteria in a demographically similar rural cohort (median age 60 vs 57, % primary educated 52.4 vs 64.0) but with substantially lower cART treatment rates (95.5% vs 49.3%) (Nyirenda et al. 2013). ICD-10 includes more somatic symptoms than DSM-IV, potentially resulting in over-reporting in chronic disease (Almeida and Almeida 1999).

Other high-income (USA) (Balderson et al. 2013; Grov et al. 2010) and middle-income settings (Brazil) (Filho et al. 2012) report prevalence of 39.1% (CES-D), 14.0% (DSM-IV), and 34.6% (GDS), respectively. These are socio-demographically different to this Tanzanian cohort (USA median age 54–55.8, %female 28–28.9, completed high school 78.7–82%) (Balderson et al. 2013; Grov et al. 2010) (Brazil mean age 57.6, %female 44.2, %literate 76.9, cART-treated 94.2%) (Filho et al. 2012).

These studies, like ours, report a high depression prevalence (highest in higher-income settings) despite differing in population demographics and proportion receiving cART. This is surprising as we might expect a higher depression prevalence among cART-untreated individuals given the biological inflammatory pathway hypothesis (Bhatia and Munjal 2014).

Baseline depression did not correlate with the HIV disease severity variables we investigated in contrast to cross-sectional studies which frequently report worse HIV disease outcomes and cART adherence. An East African study (Tanzania, Kenya, and Uganda, *n* = 2307, 58.3%female, median age 34–48) reported that depression was associated with 50% lower cART adherence and increased HIV viral load (Meffert et al. 2019). This older cohort may be unusual given that almost all received cART (vs 68% in the East African study), and self-reported adherence was high (Meffert et al. 2019). We may be observing a ‘healthy survivor’ effect which may be present in other older PLWH cohorts.

### Prevalence of other psychiatric disorders

There are few studies reporting prevalence of non-mood psychiatric disorders in HIV, particularly in low- and middle-income countries, and we were unable to identify other studies of psychiatric disorders in older PLWH in SSA. Comparisons with other studies are therefore challenging.

Psychiatric disorders other than depression were low which is consistent with similar findings in the Global Burden of Disease survey (anxiety disorders 3.4% alcohol disorders 1.4% (The Institute for Health Metrics and Evaluation 2016)). SSA studies of younger PLWH report higher levels of both anxiety and alcohol disorders. A South African study (*n* = 65, md age 30) reported a MINI DSM-IV generalised anxiety disorder prevalence of 6.7% and 10.1% alcohol dependence(Olley et al. 2006), whereas 21.7% met anxiety disorder criteria in a larger Nigerian study (*n* = 300, median age 37) (Olagunju et al. 2012).

Though our cohort included individuals in ‘middle age’, the skew towards older age may be relevant. Anxiety disorders peak in middle age and decrease in older age (Bandelow and Michaelis 2015). Similarly, alcohol misuse peaks at age 25–34 in South African general population data (Trangenstein et al. 2018).

Social circumstances may also be important. Much of our cohort were employed (88.5%) and lived with others (83%) compared to the younger South African cohort where a third lacked family support and unemployment rates were high (24.7%) (Olagunju et al. 2012).

The prevalence of mental disorders tends to be higher in those with chronic disease such as obesity, diabetes, heart disease, cancer, and COPD and increases with the number of chronic diseases (Prince et al. 2007; The Institute for Health Metrics and Evaluation 2016). A meta-analysis investigating mental disorders in chronic disease reported a 36.6% prevalence of anxiety and/or depression in chronic disease, compared to 4.4% in the general population, and that chronic disease increased the risk of anxiety and/or depression by 310% using DSM-V and ICD-10 criteria (Dare et al. 2019). The population prevalence of comorbid mental disorders (depression, anxiety, bipolar disorders, schizophrenia) with chronic diseases is estimated at 45.8% in SSA (Prince et al. 2007; The Institute for Health Metrics and Evaluation 2016). It is surprising therefore that this older cohort with a chronic disease burden appears to have lower levels of both depression and other mental disorders (Dare et al. 2019).

### Outcomes of depression at baseline

This is the only study we are aware of which investigates longitudinal outcomes of depression in older adults with HIV in SSA. The prevalence of depression appeared to decrease between baseline and follow-up though this was only significant by GDS and not DSM-IV criteria. Longitudinal reductions in depression have been reported in other studies of PLWH (supplementary Table 4), though these are majority male populations in high-income countries and/or intravenous drug user (IVDU) settings. One SSA study reported 8.9% reduction over 6 months, in the context of young PLWH before and after commencing cART (Olley et al. 2006). However, comparing existing data to our majority female cohort of older people stable on cART is challenging.

### Persistent depression and chronic inflammation hypothesis

At follow-up, individuals depressed at baseline were more likely to ‘screen-positive’ on the GDS (depressive symptoms) but not to meet case-level DSM-IV. Depression is an episodic disorder and may remit over the 2-year follow-up. However, prior episodes may increase the risk of subsequent ones, in part due to the ‘kindling’ hypothesis where the level of stressor resulting in depression is thought to have less of an effect (Rabkin et al. 1997; Johnson et al. 1999). It is unclear whether these GDS findings represent subthreshold depression.

Chronic ‘subthreshold’ depressive symptoms are common in other chronic inflammatory diseases and also in conditions resulting in damage to frontal pathways (e.g. cerebrovascular disease) commonly reported to occur in chronic HIV infection (Harrison 2017). Increased CNS and peripheral inflammatory biomarkers are reported in depression and may reduce with antidepressant medication (Hegdahl et al. 2016).

### HIV disease severity

We hypothesised that depression could be associated with ongoing HIV-related inflammation in this cohort, but baseline depression was not significantly associated with HIV disease severity including viral suppression at follow-up. In contrast, other cross-sectional SSA studies report associations with poor medication adherence, high HIV viral load, and low CD4 count (Ammassari et al. 2004; Olisah et al. 2015). This again may reflect the ‘healthy survivor’ effect and good HIV management seen in this cohort where cART adherence and HIV disease outcomes improved. HIV viral load testing introduction in 2017 may have contributed to this finding, as proportionally, more individuals receiving second-line therapy (indicating treatment failure) increased, and cART adherence also improved, potentially due to improved disease monitoring and clinician feedback.

Lack of association with HIV viral load does not exclude ongoing chronic inflammation as shown by an increased proportion receiving second-line therapy at follow-up. Chronic inflammation resulting from HIV infection may lead to depression (as with other chronic inflammatory conditions) despite a peripherally suppressed HIV viral load, given that the CNS is a ‘reservoir site’ for the HIV virus, due to limited CNS cART penetration (Gray et al. 2014). This potential ongoing CNS inflammation and damage might explain the association of depression with poorer neurological outcomes but not the measured HIV disease outcomes.

There is a high percentage of viraemic individuals at follow-up despite good reported cART adherence, and a larger proportion classified WHO stage 3 or 4 (advanced disease). Several factors may explain this finding. Some evidence suggests older age is hypothesised to increase risk of higher viral load and incomplete immune recovery (Pirrone et al. 2013). Although self-reported medication adherence is high, this may not represent objective adherence. Current guidelines suggest enhanced adherence counselling for low-level viraemia, and since viral load testing became available only a short while before our follow-up phase, many individuals may have undergone counselling prior to a switch to second-line therapy. Additionally, treatment failure may have contributed to this outcome, but we lack data on local resistance patterns.

### Neurological impairment

Baseline depression was significantly associated with self- reported neurological and functional impairments at follow-up. It is unclear whether this represents a true increase.

Somatic manifestations of depression are common in older people and in SSA (Grover et al. 2019; Lee et al. 2008). Limited access to mental health services in SSA may result in misdiagnosis of depression as physical illness (Lee et al. 2008). Self-reported ‘slow hand movements’ could represent residual depression symptoms (psychomotor retardation, anergia), somatic representations of distress, or neurological impairment.

Similarly, ‘negative cognitions’ are well-recognised in depression and may persist despite remission, potentially leading depressed individuals to over-report symptoms and impairment.

Self-reported neurological symptoms were associated with depression but the (potentially more objective) clinical diagnosis of HAND. In PLWH, depressive symptoms, but not objective neuropsychological performance, may explain the variance between self-reported and clinical findings (Rourke et al. 1999). Furthermore, self-reported functional impairment may be inaccurate in those with cognitive impairment (Thames et al. 2011). However, over-reporting is unlikely, as baseline neurological and functional impairments were not associated with depression, and other psychiatric diagnoses were uncommon. The significant follow-up associations were self-reported and clinician-rated and included the objective measure of employment. This suggests that negative cognitive biases associated with depression are not the major explanation for our findings.

### Psychosocial factors

Given that we found no association between depression and HIV disease factors, psychosocial factors may be relevant. Multiple factors such as isolation, discrimination, stigma, and living with chronic disease have been linked to depression in HIV (Munoz-Moreno et al. 2009). At baseline, we found no association between living alone, unemployment, educational background, and depression, but depression symptoms (GDS-15 ≥ 5) were associated with unemployment at follow-up.

Self-reported significant negative life events at follow-up were more likely in those depressed at baseline though overall depression prevalence reduced. Significant life stress is well-recognised to predict depression (Munoz-Moreno et al. 2009). Our findings suggest a possible bidirectional relationship. As discussed for neurological impairment, events may have been viewed more negatively due to negative cognitive biases (Wenzlaff and Bates 1998).

HIV is well-recognised to negatively impact long-term social and economic outcomes for families (Talman et al. 2013). Kenyan and South African data suggest earnings are lower in PLWH and that reduced earning potential results in sale of valuable assets such as livestock causing ongoing health and economic vulnerability (Talman et al. 2013). PLWH with depression may also be more likely to experience disability and reduced work productivity. In India, depression in HIV was associated with poorer environmental and social quality of life indicators (unemployment, low-income, poor diet, inadequate housing, and lack of life partner) (Charles et al. 2012). Similarly, a Ugandan study reported PLWH with depression (PHQ-9 and MINI) to be more likely to be unemployed and/or have lower weekly income than those without depression (Wenzlaff and Bates 1998). Therefore, depressed PLWH may be at higher risk of negative socioeconomic life events (Deshmukh et al. 2017).

Individuals with depression may be more likely to experience difficult social situations, negative interactions, and selectively focus on negative emotional stimuli (Steger and Kashdan 2009). This may result in fewer intimate relationships, greater negative responses from others, and greater emotional distress in response to social stressors (Steger and Kashdan 2009). Consequently, PLWH with depression may be more likely to experience stressful life events and also respond more negatively to them, creating a double burden of disability, whilst having lower ‘reserve’ due to lower socioeconomic status (Wagner et al. 2012). Conversely other studies report lower subsequent negative life events and stressors at follow-up in those with depression (Olley et al. 2006).

In summary, though comparable longitudinal data are lacking, it seems plausible that both HIV and depression may interact to result in greater self-reported negative life events and worse functional outcomes (‘objective’ and self-reported). Our data indicates that inflammation from HIV onto the CNS and physical stressors work together to manifest depression in PLWH.

### Limitations

We selected a local translation of the GDS-15 based on extensive use in SSA but recognise that limitations in transcultural settings are well-recognised (Howorth et al. 2019). Similarly, we used a local translation of the MINI, previously used in the same clinic (Sumari-de Boer et al. 2018), but the low observed prevalence of some psychiatric disorders could be attributable to cultural differences in understanding of the MINI questions. We nevertheless could not identify any robustly validated cultural alternative. Though we report on psychosocial outcomes, we did not obtain information on self-perceived stigma and socioeconomic background, risk, and outcome factors which may have been relevant.

At baseline, psychiatric screening was completed by registered Tanzanian nurses with mental health research experience (one at doctoral level). Despite efforts to develop rapport, sensitivity of the questions and well-recognised stigma associated with mental illness may have led to under-reporting of psychiatric symptoms. Similarly, self-reported negative life events may have been under- or over-reported depending on rapport. Due to scarcity of specialist mental health services in Tanzania, previous psychiatric disorders are likely to be undiagnosed and subsequently under-reported. Similarly, since depression is episodic, depression which was not present during the study period will have been missed.

In 2018, the psychiatric interview was abbreviated to prevent participant fatigue and retain as many participants as possible. Since there were low rates of other psychiatric disorders, we decided to focus on depression. This may have impacted longitudinal findings.

Neurological symptoms were self-reported. A neurological examination was completed at baseline and follow-up but was insufficiently comprehensive to fully corroborate symptoms (e.g. peripheral neuropathy). The lack of HIV viral load testing at baseline limited assessment of HIV disease severity.

We had a relatively high rate of loss to follow-up. However, we were able to verify from clinic records that almost all not evaluated (72/91) remained under active HIV clinic follow-up and only 8/91 were recorded to have died. Though those seen and not seen at follow-up did not differ in recorded sociodemographic factors, it is not clear whether the follow-up cohort was representative in terms of outcome. Individuals with more severe depression may be less likely to engage with medical services, potentially skewing our results (DiMatteo et al. 2000; Dixon et al. 2016).

Study data collection corresponded with the crop-planting (rainy) season. Those failing to attend clinic may have been fitter and prioritising the annual community agricultural activities or, conversely, less able to travel in difficult road conditions.

## Conclusion

This is the first longitudinal study of prevalence and outcomes of depression in older PLWH in SSA. Despite well-controlled disease, significant associations were found with neurological and functional impairment and an increased risk of subsequent negative significant life events. This suggests that depression may have long-lasting effects despite remission. This cohort, demographically typical of PLWH in Tanzania, markedly differs from high-income studies of older PLWH. There are likely to be differing aetiologies and risk factors for depression that are not currently understood. Further research should replicate these findings in SSA; identify those at greatest risk; and determine whether interventions can reduce these observed negative outcomes.

## Supplementary Information

Below is the link to the electronic supplementary material.

Supplementary file1 (DOCX 2270 KB)

## Data Availability

The authors confirm that deidentified data supporting the findings of this study are available upon reasonable request to the corresponding author, where necessary subject to a data transfer agreement and permission from the Tanzanian National Institute for Medical Research (NIMR).
