# Supplementary material for: The prevalence and outcomes of depression in older HIV-positive adults in Northern Tanzania: a longitudinal study
Source: J Neurovirol. 2023 May 25;29(4):425–39. doi: 10.1007/s13365-023-01140-4 (PMC10501928; doi:10.1007/s13365-023-01140-4)
Supplement: Supplementary file 1 — Supplementary file1 (DOCX 2270 KB) [file 13365_2023_1140_MOESM1_ESM.docx]

*Supplementary Table 1 – Self-Report Neurological Questionnaire*

| **Initial Screen** | 1=yes, 2=no, 3=don’t know, 9=missing | If yes, please describe your problems here: e.g. Further details of low mood etc. |
| --- | --- | --- |
| **B.3.1** Do you think you have problems with your memory? | **a.** | **b.** |
| **B.3.2** Do you have difficulty with concentration, this means doing a task which takes a while. Are you able to pay attention to a sermon at the church or mosque, listen to a whole programme on the radio/news? | **a.** | **b.** |
| **B.3.3** Do you have difficulty thinking clearly or making decisions? | **a.** | **b.** |
| **B.3.4** Do you have difficulty with your balance or falling? | **a.** | **b.** |
| **B.3.5** Do you have difficulty with slow hand movements? | **a.** | **b.** |
| **B.3.6** Do you have loss of feeling in your hands or feet? | **a.** | **b.** |
| **B.3.7** Do you have headaches? | **a.** | **b.** |
| **B.3.8** Do you have difficulty in carrying out your normal day to day work?  Please describe your difficulties here | **a.** | **b.** |
| **B.3.9** Do you have difficulty in carrying out your home responsibilities? Please describe any difficulties or problems here | **a.** | **b.** |

*Supplementary Table 2: Baseline (2016) demographic and HIV-disease data (n=253)*

| **Sex** (female) | 183 (72.3%) |
| --- | --- |
| **Age** (md, range) | 57 (50-79) |
| **Education** (primary school educated) | 162 (64.0%) |
| **Unemployed** (missing = 6) | 29 (11.5%) |
| **Lives Alone** (missing = 4) | 43 (17.0%) |
| **Current CD4 count** (mm/l) mn, SD | 526.5(255.2) |
| **Nadir CD4 count** (mm/l) mn, SD | 197.2 (160.7) |
| **Medication adherence** (100% adherence) | 175 (69.2%) |
| **Tuberculosis** | Current Infection: 5 (2.0%)  Previous Infection: 42 (16.6%) |
| **Empirical central nervous system infection treatment** | Current: 8 (3.2%)  Previous: 18 (7.1%) |
| **BMI** (mn, SD) | 22.65 (4.59) |
| **cART Regimen**  (missing = 17) | First-line: 211 (83.3%)  Second line: 25 (9.9%) |
| **On cART** | 242 (95.5%) |
| **Efavirenz** (missing = 8) | 134 (53.0%) |
| **WHO Stage**  (missing = 69) | 1-2: 32 (12.6%)  3-4: 152 (60.0%) |
| **Prevalence of Depression by GDS criteria** | 53 (20.9%) |
| **Prevalence of Depression by DSM-IV criteria** | 42 (16.6%) |

*Supplementary Table 3: Demographic Characteristics of Follow-Up Cohort (those seen in 2016 and 2018, n=162)*

|  | **2016** | **2018** |
| --- | --- | --- |
| **Sex (female)** | 183 (72.3%) | 114 (70.4%) |
| **Age (md, range)** | 57 (50-79) | 59  (52-81) |
| **Education** |  |  |
| Primary School not completed | 91 (36.0%) | 52 (32.1%) |
| Primary school educated | 162 (64.0%) | 110 (67.9%) |
| **Unemployed**  (missing = 6) | 29 (11.5%) | 16 (9.9%) |
| **Lives Alone**  (missing = 4) | 43 (17.0%) | 37 (22.8%) |
| **Current CD4 count** (mm/l) |  |  |
| Mean | 526.5 | 530.18 |
| SD | 255.2 | 255.17 |
| **Nadir CD4 count** (mm/l) |  |  |
| Mean | 197.2 | 203.01 |
| SD | 160.7 | 167.88 |
| **Self-reported Medication Adherence**  (100% adherence) | 175 (69.2%) | 137 (84.6%) |
| **Tuberculosis** |  |  |
| Current Infection | 5 (2.0%) | 0 (In previous 12 months) |
| Previous Infection | 42 (16.6%) |  |
| **Central Nervous System** |  |  |
| Current Infection | 8 (3.2%) | 0 (In previous 12 months) |
| Previous Infection | 18 (7.1%) |  |
| **BMI** |  |  |
| Mean | 22.65 | 22.8 |
| SD | 4.59 | 4.9 |
| **ART Regimen** |  |  |
| First-line | 211 (83.3%)  (missing = 17) | 84 (51.9%)  (missing = 61) |
| Second Line | 25 (9.9%) | 17 (10.5%) |
| **On ART** | 242 (95.5%) |  |
| **Efavirenz** | 134 (53.0%)  (missing 8) | 51 (31.5%)  (missing = 3) |
| **WHO Stage** |  |  |
| 1-2 | 32 (12.6%) | 24 (14.8%) |
| 3-4 | 152 (60.0%)  (missing = 69) | 127 (93.2%)  (missing = 11) |
| **Viral Load Suppressed** | Data not available in 2016 | 110 (67.0%)  (missing = 11) |
| **Psychiatric Medications**  (amitriptyline) | 7 (2.8%) | 6 (3.7%) |
| **Prevalence of Depression by GDS criteria** | 53 (20.9%) | 21 (13.0%) |
| **Prevalence of Depression by DSM-IV criteria** | 42 (16.6%) | 18 (11.1%) |

*Supplementary Table 4: Studies Investigating the Longitudinal Prevalence of Depression in People Living with HIV*

| **Author** | **Country** | **Population** | **Prevalence at Baseline** | **Prevalence at follow- up** | **Mean Age** | **% on ART** | **Depression Criteria** | **Length of follow up** |
| --- | --- | --- | --- | --- | --- | --- | --- | --- |
| (Rodkjaer et al., 2011)([Rodkjaer et al. 2011](#_ENREF_49)) | Denmark | Adults – general population | 26.0% | 16.0% | Data not available | 80.0 | Beck Depression Inventory II | 3 years |
| (Olley et al., 2006)([Olley, Seedat, and Stein 2006](#_ENREF_40)) | South Africa | Recently diagnosed adults | 34.9% | 26.0% | 30 | 1.3 | MINI | 6 months |
| (Johnson et al., 1999)([Johnson et al. 1999](#_ENREF_28)) | USA | IVDU | 18.1% | 16.2% | 39 | Data not available | Structured Clinical Interview for DSM-III-R | 3 years |
| (Orlando et al., 2002)([Orlando et al. 2002](#_ENREF_43)) | USA | Adults – general population | 22.0% | 18.6% | Data not available | Data not available | CIDI-SF and CIDI (DSM-IV criteria) | 8 months |
| (Rabkin et al., 1997)([Rabkin et al. 1997](#_ENREF_48)) | USA | IVDU | 26.0% | 14.0% | 38.5 | Data not available | Structured Clinical Interview for DSM-III-R | 3 years |
